# Dealing with Schizophrenia in a crowded restaurant

**DOI:** 10.1038/s41537-022-00318-9

**Published:** 2022-12-12

**Authors:** Jason Jepson

**Affiliations:** Schizophrenia, https://www.nature.com/npjschz/

**Keywords:** Diseases, Schizophrenia

When I wake up early for a doctor’s appointment, I treat myself to breakfast at a local restaurant. It is a friendly place. Something I enjoy doing when I am in a public place is people watching. However, I must be careful because most people are not comfortable knowing that someone is staring at them.

This restaurant has a TV, so I can watch sports highlights in order to tune out the voices and paranoia that come from my schizophrenia diagnosis. Watching TV allows me to tune out those around me who I feel are reading my mind. Despite the fact I might feel that way, I still try to be alert to the server who is taking my order. I want to smile and appear to be a friendly person.

After my order is taken, I either go back to watching television or start to play with my phone. One of the positive aspects of owning a pone is that it lets me appear to be busy. I use my phone, like the television to distract myself from my symptoms. Once eating at this restaurant, I thought I heard another customer ask the server if she thought I was stalking her. I have learned while living with schizophrenia not to react to these thoughts but to find ways to distract myself.

Being part of a group in a restaurant brings about different challenges for me. When I am in a group of people at a restaurant, I make sure there is at least one person with whom I feel comfortable which helps to ease my social anxieties. Having someone who understands my situation allows me to be myself. Often, I look around the restaurant to see if the voices I hear are in my head or coming from other tables of customers. I wonder if those seated at my table or around the restaurant can actually see my anxieties. Quietly practicing controlled breathing exercises helps to calm me down when I am in a group. I am the most comfortable when I am with family members because I know they understand my diagnosis, and they know I am not being rude, but dealing with my challenges.

In a group of people, I do not feel I have to start up brilliant conversations. I do not have to be profound. I do not mind small talk, and despite my anxieties, human contact, even at a minimum, makes me feel good. Feeling part of the conversation makes me smile.

If I am asked a question, I think about it and repeat the question again in my mind. I am not afraid to answer, “I don’t know,” but I try to think of a satisfying answer.

The big issue around my belief that people are talking about me can be managed when I have to listen to real conversation that draws me in. This is real, and this is what is going on. The server might be standing in front of me asking me what I want to eat. It is hard to concentrate, but when I hear a real voice or real question, I listen, and it brings me to the reality of the moment.

I am thankful that I have the means to occasionally eat out whether alone or with a group. I tell myself the other customers do not know me, and the people sitting at my table cannot read my thoughts. I am there for a meal and friendly conversation, and I am the one paying for my meal.

